# Soil Water Content Shapes Microbial Community Along Gradients of Wetland Degradation on the Tibetan Plateau

**DOI:** 10.3389/fmicb.2022.824267

**Published:** 2022-02-02

**Authors:** Meng Li, Kerou Zhang, Zhongqing Yan, Liang Liu, Enze Kang, Xiaoming Kang

**Affiliations:** ^1^Institute of Wetland Research, Chinese Academy of Forestry, Beijing, China; ^2^Beijing Key Laboratory of Wetland Services and Restoration, Beijing, China; ^3^Sichuan Zoige Wetland Ecosystem Research Station, Tibetan Autonomous Prefecture of Aba, China; ^4^National Disaster Reduction Center of China, Ministry of Emergency Management, Beijing, China; ^5^Satellite Application Center for Disaster Reduction, Ministry of Emergency Management, Beijing, China

**Keywords:** alpine wetland, ecosystem degradation, microbial networks, soil physicochemical properties, 16S rRNA sequencing, microbial community composition

## Abstract

Soil microbes are important components in element cycling and nutrient supply for the development of alpine ecosystems. However, the development of microbial community compositions and networks in the context of alpine wetland degradation is unclear. We applied high-throughput 16S rRNA gene amplicon sequencing to track changes in microbial communities along degradation gradients from typical alpine wetland (W), to wet meadow (WM), to typical meadow (M), to grassland (G), and to desert (D) in the Zoige alpine wetland region on the Tibetan Plateau. Soil water content (SWC) decreased as wetland degradation progressed (79.4 and 9.3% in W and D soils, respectively). Total organic carbon (TOC), total nitrogen (TN), and total phosphorus (TP) increased in the soils of WM, and then decreased with alpine wetlands degradation from WM to the soils of M, G, and D, respectively. Wetland degradation did not affect microbial community richness and diversity from W soils to WM, M, and G soils, but did affect richness and diversity in D soils. Microbial community structure was strongly affected by wetland degradation, mainly due to changes in SWC, TOC, TN, and TP. SWC was the primary soil physicochemical property influencing microbial community compositions and networks. In wetland degradation areas, *Actinobacteriota*, *Acidobacteriota*, *Cholorflexi*, and *Proteovacteria* closely interacted in the microbial network. Compared to soils of W, WM, and M, *Actinobacteriota* played an important role in the microbial co-occurrence network of the G and D soils. This research contributes to our understanding of how microbial community composition and networks change with varied soil properties during degradation of different alpine wetlands.

## Introduction

Soil microbes play a vital role in many ecological processes of wetlands and are essential for maintaining ecosystem functions and stability (Wu et al., 2021). The microbial community is closely associated with ecological functioning of soil microbes, because of the diverse functions provided by the different microbial species (Crowther et al., 2019). The diversity of microbial communities has a positive impact on element cycling, nutrient uptake, organic matter decomposition, and toxin removal (Tang et al., 2011; Wu et al., 2015). The microbial community also contributes to soil structure regulation, soil formation and plant productivity (Zhang et al., 2013; Gao et al., 2021). The soil microbial community is sensitive to variation in soil physicochemical properties and can serves as an indicator of soil quality (Yu et al., 2012; Wu et al., 2021). In reverse, changes in microbial community can also provide feedback to ecosystem functions and may offer an opportunity to mitigate the impact of ecosystem alterations induced by human disturbance and environmental variation (Huang et al., 2020).

Wetlands are generally classified as a transitional ecosystem between aquatic and terrestrial ecosystems. They play a unique ecological role in the maintenance of biodiversity (Aber et al., 2012; Ren et al., 2013; Lin et al., 2021). Wetlands function as the “kidney of the Earth” by providing the ecological services of nutrient cycling, pollutant filtration, flood abatement, and carbon sequestration (Tang et al., 2011; Zhang et al., 2013). Wetlands cover 6% of the world’s terrestrial surface area, but have a total carbon storage of 770 billion tons. This accounts to 35% of the total carbon stored in terrestrial ecosystems (Meng et al., 2017; Zhang et al., 2022). However, over 50% of global wetlands have suffered threatens from human activity and climate change, and this has caused substantial declines in their area and in the degradation of ecosystem functions (Sica et al., 2016; Jiang et al., 2017; Khaledian et al., 2017). Previous studies have revealed that the wetland degradation may lead to a succession of wetland ecosystems, such as marsh degradation into meadow, grassland, and even desert (Ren et al., 2013; Wu et al., 2015). Wetland ecosystem degradation can occur along with water table drawdown, changes in soil properties, vegetation degradation, and soil organic matter mineralization (Wu et al., 2021). Soil microbes are strongly associated with the hydrological and soil biogeochemical processes that occur during wetland degradation (Wu et al., 2021). Wetland degradation may modify soil and microbial performance (Ren et al., 2013). In the alpine wetland of the Yellow River source zone in western China, soil total organic carbon (TOC) and total nitrogen (TN) decreased following wetland degradation (Li H. et al., 2021; Lin et al., 2021). An increase in soil pH was also observed in the Jiuduansha wetlands along with the degradation stages (Tang et al., 2011). Changes in soil pH, moisture, and nutrient status caused by wetland degradation may alter microbial diversity and assemblies (Wu et al., 2015, 2021; Zhao et al., 2021).

There is a total area of 13.19 × 10^4^ km^2^ of alpine wetland across the Tibetan Plateau. This region is crucial for water conservation and climate regulation in China (Wu et al., 2015; Yan et al., 2021). Alpine wetlands are fragile ecosystems characterized by high altitudes and low temperatures. They harbor unique alpine biodiversity, which is at a high risk of degradation due to climate change, drainage, and overgrazing (Wu et al., 2015; Lin et al., 2021). Degradation on the Tibetan Plateau can be followed by large-scale wetland ecosystem succession, which is often accompanied by a degradation gradient from typical wetland to wet meadow, to typical alpine meadow, to grassland, and sometimes to desert (Ren et al., 2013; Wu et al., 2015; Shen et al., 2019). The degradation of the alpine wetland often involves a hydrological gradient that includes perennial flooded, water-saturated, humid, semi-humid, and arid (Ren et al., 2013; Lin et al., 2021). In alpine wetlands, soil microbial community has a relative stability for a resistance to stress of environmental changes, but long-term and high-intense wetland degradation may beyond the capacity and induce changes in microbial community (Li Y. et al., 2021). Many studies have examined the responses of soil nutrients, physicochemical properties, enzyme activity, plant communities, and microbial diversity to degradation of alpine wetlands (Li Y. et al., 2021). However, a comparison of soil microbial community compositions and networks at different stages of degradation has not yet been conducted. The understanding of microbial community compositions and networks as affected by alpine wetland degradation may provide a technique basis for regulating microbial community to restore wetland degradation. Therefore, we investigated the changes in soil microbial community compositions and networks associated with degradation of alpine wetlands to determine: (1) the key microbial communities affected by wetland degradation and their co-occurrence interactions, and (2) correlation of soil properties with the changes in microbial communities and networks.

## Materials and Methods

### Study Area and Soil Sampling

**FIGURE 1:**
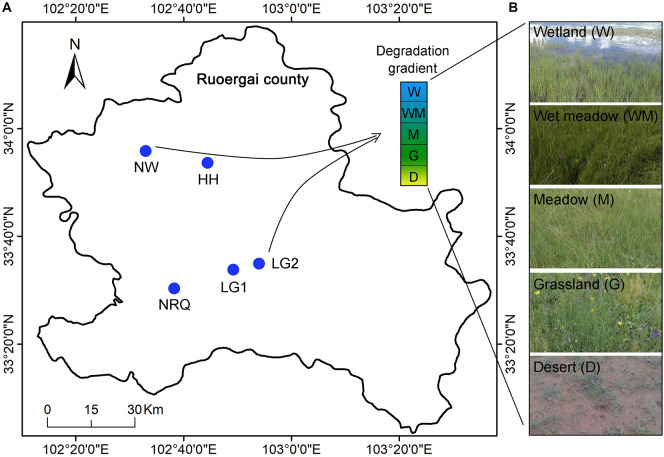
Map of sampling **(A)** and vegetation **(B)** along degradational gradients of alpine wetland at Neng Wa (NW), Hua Hu (HH), Na Ruoqiao (NRQ), and Ling Ga (LG1 and LG2). W, typical wetland; WM, wet meadow; M, meadow; G, grassland; D, desert.

The study area is located in the Zoige alpine wetland, Ruoergai County in the northeastern part of the Tibetan Plateau. The Zoige wetland is the largest plateau peatland around the world, with a total area of 16,670.6 km^2^ and an average elevation of 3,500 m. The Zoige region has a humid monsoon climate in the cold-temperate plateau zone, with a mean annual precipitation of 600–750 mm and a mean annual temperature of 0.96°C (Jiang et al., 2017). At the five sites in the study area Na Ruoqiao (NRQ), Neng Wa (NW), Ling Ga (LG1 and LG2), and Hua Hu (HH), sampling plots were selected with five gradients of wetland degradation at each site: typical wetland (W), wet meadow (WM), meadow (M), grassland (G), and desert (D) (Figure 1). The five sites were distributed within 30 km each other in the Zoige region. They had the same climate, similar elevations of 3,394–3,466 m, and the same peat soil parent material. In each plot, three surface soils (0–20 cm) were randomly collected and mixed into one soil sample. A total of 25 soils were sampled, stored at –20°C, and quickly transported to the laboratory. One part of the soil sample was air-dried, and ground to pass through 0.15-mm sieve for the characterization of basic properties, and the other fresh part was purified by removal of root and gravel and was then used for the analysis of the microbial community and microbial biomass P (MBP).

### Soil Characterization

Soil water content (SWC) was measured by the weight difference before and after oven-drying at 105°C. Soil pH was determined using a pH meter (FE20-FiveEasy Plus, Mettler Toledo, Switzerland) in a 1:2.5 water/soil ratio. Soil total organic carbon (TOC) and total nitrogen (TN) were determined by the H_2_SO_4_–K_2_Cr_2_O_7_ wet oxidation and the Kjeldahl methods, respectively (Bremner and Mulvaney, 1982; Pribyl, 2010). Dissolved organic carbon (DOC) was extracted by deionized water in a soil/water ratio of 1:5 and detected with a total organic carbon/TN analyzer (Multi C/N 2100, Analytik Jena, Germany) (Jones and Willett, 2006). Soil was digested by HF-H_2_O_2_–HNO_3_ in a microwave system (MARS 5, CEM, United States) at 180°C for 30 min. Total phosphorus (TP) was then analyzed by inductively coupled plasma-optical emission spectroscopy (ICP-OES, Optima 8000, Perkin Elmer, United States) (Li et al., 2015). MBP was determined by fumigation extraction (Zederer et al., 2017).

### DNA Extraction and 16S rRNA High-Throughput Sequencing

Soil DNA was extracted from approximately 0.5-g fresh soil sample using FastDNA Spin kits (MP Biomedicals, United States) following the manufacturer’s instructions. A NanoDrop 2000 spectrophotometer (Thermo Fisher Scientific, Waltham, MA, United States) and a QuantiFluor dsDNA system (Promega, United States) were used to detect the purity and content of the extracted DNA. The primers 338F (5’-ACTCCTACGGGAGGCAGCAG-3’) and 806R (5’-GGACTACHVGGGTWTCTAAT-3’) were applied to amplify the V3–V4 hypervariable regions of the 16S rRNA genes by a previously described PCR amplification procedure (Hu et al., 2019). The PCR amplicons were separated on a 2% agarose gel and purified using an AxyPrep DNA gel extraction kit (Axygen Biosciences, United States). Paired-ends of the purified amplicons were then sequenced on a Miseq 300 platform (Illumina, United States).

The 16S rRNA sequencing data were processed using the QIIME 1.9.1 pipeline. Operational taxonomic units (OTUs) were clustered at the 97% sequence similarity threshold using Uparse v7.0.1090 and were assigned to the Silva 138 database using the RDP Classifier v2.11. Alpha diversity indices of Shannon, Chao, Ace, Simpson, and Good’s non-parametric coverage estimator were calculated using Mothur v1.30.2 to estimate the diversity and richness of the microbial community.

### Statistical Analysis

One-way analysis of variance (ANOVA) was used to test the differences of basic soil properties and microbial taxa between different degradation gradients using the “aov” package in R v4.1.1. Redundancy analysis (RDA) was conducted using the “vegan” package in R v4.1.1 to detect the multivariate relationships between soil properties and microbial community under different degradation gradients (Dixon, 2003). A heatmap describing the correlation between soil properties and the main microbial phyla was generated using the “pheatmap” package in R v4.1.1 (R core team, 2019). Spearman’s rank correlation based on the false discovery rate correction was performed to assess the association among microbial OTUs from all the soils. One-way microbial co-occurence networks across five degradation gradients were built using Cytoscape v3.8.2 to evaluate the complexity of the microbial community and interconnection among the microbial taxa. Top 500 dominant OTUs accounting for 68.81% of relative abundance were included to construct the networks and were shown as nodes in the networks. Correlation between different microbial taxa with Spearman’s correlation coefficient (*r*-value) > 0.8 at significant level *P* < 0.01 were shown as the edges in the networks and the weight of each correlation is proportional to edge thickness. The nodes were colored according to microbial phyla and node size is proportional to the sum of each OTU in all the soils. Relevant topological parameters of the resulting networks including numbers of nodes and edges, average number of neighbors, degree, and closeness centrality were then obtained. High closeness centrality and high degree for all the investigated OTUs was ranked to identify the keystone taxa (Wan et al., 2020; Zheng et al., 2021). A two-way network was also constructed using Cytoscape v3.8.2 to examine the microbial network as affected by soil properties of SWC, TOC, and MBP. Correlation between microbial taxa and soil properties with Spearman’s correlation coefficient (*r*-value) > 0.5 at significant level *P* < 0.01 were used for the construction of two-way network.

## Results

### Basic Soil Properties

SWC decreased along the wetland degradation from 79.4% on average in the W soils to 56.2% in the WM soils, 40.7% in the M soils, 29.4% in the G soils and 9.3% in the D soils (Table 1). Soil pH ranged from 5.87 to 8.29 with an average of 7.03, and most of the soils were neutral or weakly alkalic. In addition, the pH value showed no significant difference between different wetland degradation stages. The WM and D soils had the highest (143.10 ± 67.94 mg g^–1^) and lowest (3.83 ± 3.30 mg g^–1^) TOC contents, respectively. Along the wetland degradation, the DOC, TP, and MBP contents increased in the WM and M soils compared with the W soils and then decreased in the D soils when compared with the WM and M soils (Table 1). The D soils had the lowest TN content (0.31 ± 0.17 mg g^–1^) among the soils.

**TABLE 1:** Soil basic properties along alpine wetland degradation gradients.

Degradation	SWC /%	pH	TOC /mg g^–1^	DOC /mg kg^–1^	TP /mg g^–1^	TN /mg g^–1^	MBP /mg g^–1^
W	79.4 ± 3.0a	6.99 ± 0.92a	68.21 ± 44.92b	40.76 ± 21.70b	692.26 ± 183.27b	4.47 ± 2.66b	8.65 ± 1.82b
WM	56.2 ± 3.7b	7.16 ± 0.88a	143.1 ± 67.94a	71.56 ± 42.56a	1069.08 ± 198.61a	9.99 ± 3.99a	20.94 ± 7.99a
M	40.7 ± 3.8c	6.65 ± 0.70a	75.39 ± 39.99b	57.71 ± 20.43a	1172.12 ± 592.68a	8.61 ± 1.45a	18.96 ± 4.50a
G	29.4 ± 2.5d	6.91 ± 0.92a	38.37 ± 11.84c	45.02 ± 10.46ab	841.19 ± 151.17ab	3.57 ± 1.17b	17.24 ± 4.03a
D	9.3 ± 2.0e	7.41 ± 0.91a	3.83 ± 3.30d	39.63 ± 8.34b	384.83 ± 105.33c	0.31 ± 0.17c	5.01 ± 2.36c

*W, typical wetland; WM, wet meadow; M, meadow; G, grassland; D, desert.*

*SWC, soil water content; TN, total nitrogen; TOC, soil total organic carbon; DOC, dissolved organic carbon; TP, total phosphorus; MBP, microbial biomass phosphorus. Different letters represent significant difference at P < 0.05.*

### Diversity Indices of the Microbial Community Under Different Degradation Gradients

**FIGURE 2:**
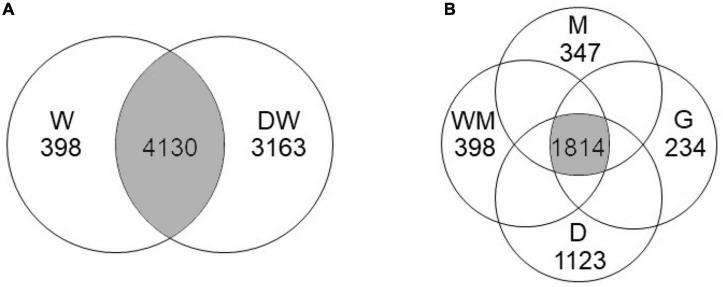
Venn diagrams of core operational taxonomic units (OTUs) shared **(A)** between wetland (W) and degraded wetland [DW, including wet meadow (WM), meadow (M), grassland (G), and desert (D)], and **(B)** among WM, M, G, and D.

A total of 898,876 qualified reads were obtained by 16S rRNA sequencing from 25 fresh soils with an average length of 418 bases. Based on a species similarity of 97%, a total of 7,691 OTUs were clustered with the average Good’s coverage of 0.973. No significant difference in the number of OTU number was found among the W, WM, M and G soils (Table 2). The D soils had the smallest number of OTUs number (1751 on average). Among the total OTUs, the W and the DW soils shared 4,130 OTUs, and the DW had 3,163 unique OTUs (Figure 2). The M, WM, G, and D soils shared 1,814 OTUs, and the D soils had the highest number of unique OTUs (1,123). The Shannon, Simpson, Ace, and Chao indices of the microbial community in the soils were 5.74–6.50, 0.0038–0.0139, 2094.4–3356.6, and 2068.5–3325.6, respectively (Table 2). No significant differences in these four indices were found among the W, WM, M, and G soils. The D soils had the lowest Shannon (6.06 ± 0.18), Ace (2382.5 ± 254.6), and Chao (2395.8 ± 237.8) of microbial community indices.

**TABLE 2:** Microbial richness and diversity indices for clustering at 97% identity in soils along wetland degradation gradients.

Degradation	OTU number	Shannon	Simpson	Ace	Chao	Coverage
W	2002 ± 263a	6.18 ± 0.19a	0.0067 ± 0.0013a	2625.6 ± 398.9a	2597.2 ± 402.2a	0.972 ± 0.005a
WM	1966 ± 176a	6.19 ± 0.15a	0.0059 ± 0.0015a	2655.8 ± 263.9a	2615.9 ± 277.7a	0.971 ± 0.003a
M	1931 ± 300a	6.18 ± 0.28a	0.0056 ± 0.0023a	2621.7 ± 402.3a	2595.4 ± 390.2a	0.971 ± 0.004a
G	2065 ± 226a	6.37 ± 0.05a	0.0055 ± 0.0011a	2663.0 ± 523.3a	2634.4 ± 512.9a	0.973 ± 0.008a
D	1751 ± 156b	6.06 ± 0.18b	0.0071 ± 0.0038a	2382.5 ± 254.6b	2395.8 ± 237.8b	0.974 ± 0.003a

*OTU, operational taxonomic unit; Shannon, Shannon-Weiner index.*

*W, typical wetland; WM, wet meadow; M, meadow; G, grassland; D, desert.*

*Different letters represent significant difference at P < 0.05.*

### Microbial Community Compositions Under Different Degradation Gradients

**FIGURE 3:**
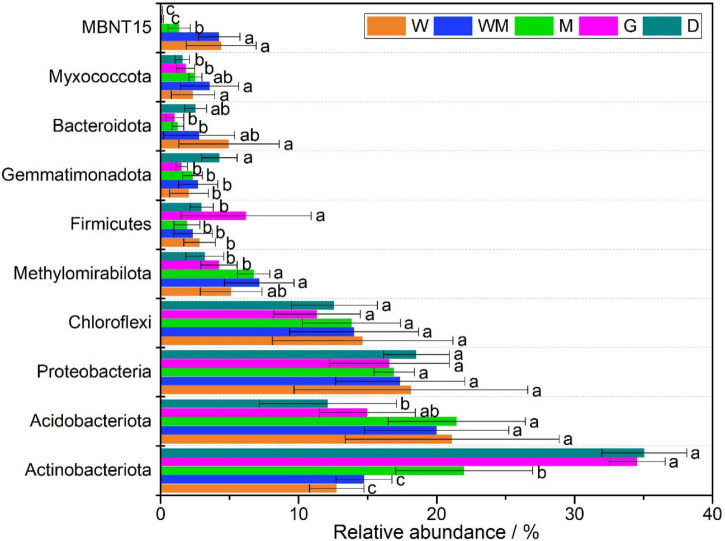
Relative abundance of the top 10 phyla along alpine wetland degradation gradients. W, typical wetland; WM, wet meadow; M, meadow; G, grassland; D, desert. Different letters indicate significant difference at *P* < 0.05.

The obtained sequencing reads were assigned to 55 phyla and 1,093 genera. The top ten dominant phyla were *Actinobacteriota* (10.6–39.1%), *Acidobacteriota* (7.1–31.4%), *Proteobacteria* (9.2–28.9%), *Chloroflexi* (7.9–22.6%), *Methylomirabilota* (1.0–11.5%), *Firmicutes* (0.9–14.2%), *Gemmatimonadota* (0.3–5.6%), *Bacteroidota* (0.4–10.6%), *Myxococcota* (0.6–6.3%), and *MBNT15* (0.03–7.2%) (Figure 3). The W and WM soils had lower relative abundances of *Actinobacteriota* and higher relative abundance of *Acidobacteriota* than the D soils. The relative abundances of *Proteobacteria* and *Chloroflexi* were similar among the soils. The relative abundances of *MBNT15*, *Myxococcota*, and *Methylomirabilota* were lowest in the D soils, while the relative abundance of *Gemmatimonadota* was highest in the D soils (Figure 3).

### Relationships Between Soil Properties and Microbial Community

**FIGURE 4:**
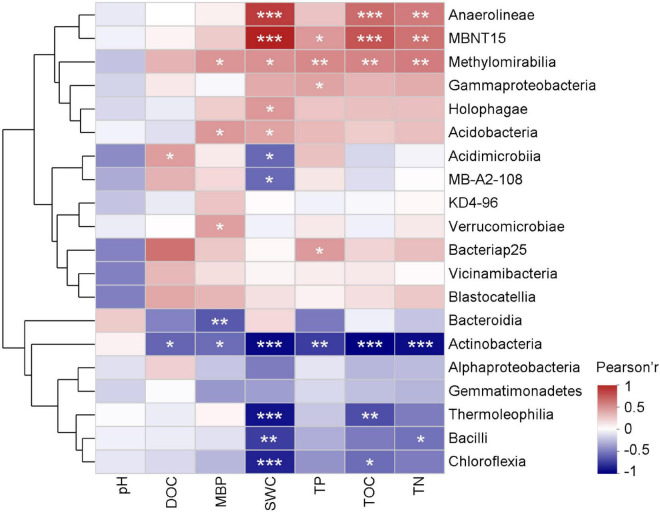
Heatmap showing the correlation between soil properties and the main microbial classes. *, **, and *** indicate significant correlations at *P* < 0.05, 0.01, and 0.001, respectively. DOC, dissolved organic carbon; MBP, microbial biomass phosphorus; SWC, soil water content; TP, total phosphorus; TOC, total organic carbon; TN, total nitrogen.

Soil pH had no significant effect on the microbial community structure (Figure 4). The heatmap clustered two groups of main microbial classes based on the negative/positive correlations between soil properties and microbial taxa. Among the top 20 microbial classes, correlations between the soil properties and relative abundances of *KD4-96*, *Vicinamibacteria*, *Blastocatellia*, *Alphaproteobacteria*, and *Gemmatimonadetes* were not significant. Relative abundance of *Actinobacteria* was negatively correlated with DOC, MBP, SWC, TP, TOC, and TN. SWC was negatively correlated with *Thermoleophilia*, *Bacilli*, *Acidimicrobiia*, *MB-A2-A08*, and *Chloroflexi*, but was positively correlated with *Anaerolineae*, *MBNT15*, *Methylomirabilia*, *Holophagae*, and *Acidobacertia*. TOC and TN showed significant positive correlations with *Anaerolineae*, *MBNT15*, and *Methylomirabilia* (*P* < 0.01).

**FIGURE 5:**
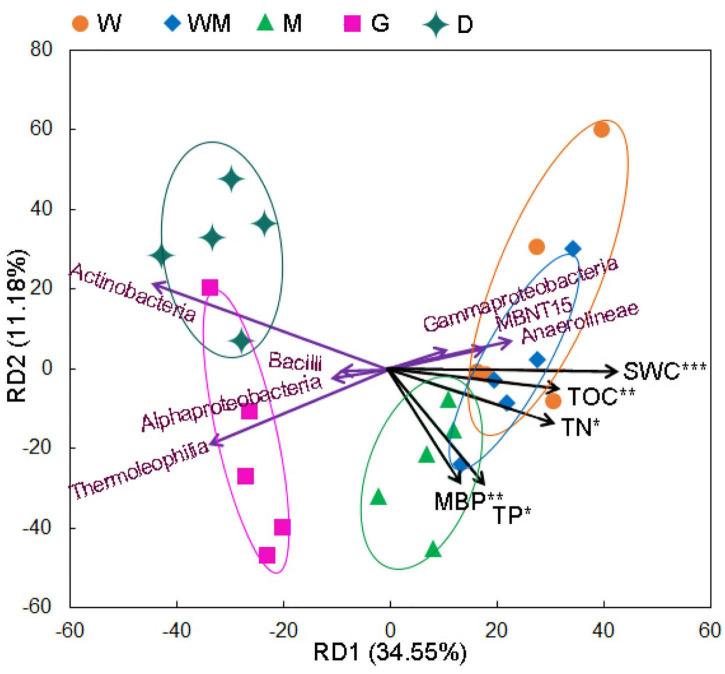
Redundancy analysis (RDA) of the multivariate relationships among soil properties and microbial classes along degradation gradients (W, typical wetland; WM, wet meadow; M, meadow; G, grassland; D, desert). MBP, microbial biomass phosphorus; SWC, soil water content; TP, total phosphorus; TOC, total organic carbon; TN, total nitrogen. *, **, and *** represent significant correlation at *P* < 0.05, *P* < 0.01, and *P* < 0.001, respectively.

RDA showed the effect of soil properties on the microbial community (Figure 5). The RD1 and RD2 explained 34.55 and 11.18% of the total variation, respectively. A distinct separation among the four groups of soil samples was found. The W soil group was not neatly separated from the WM soil group. Along the RD1 axis, lower relative abundances of *Actinobacteria* and *Thermoleophilia* in the G and D soils were mainly associated with lower SWC, TOC, and TN. Along the RD2 axis, MBP and TP may be associated with the separation of the D soil group from the G soil group.

**FIGURE 6:**
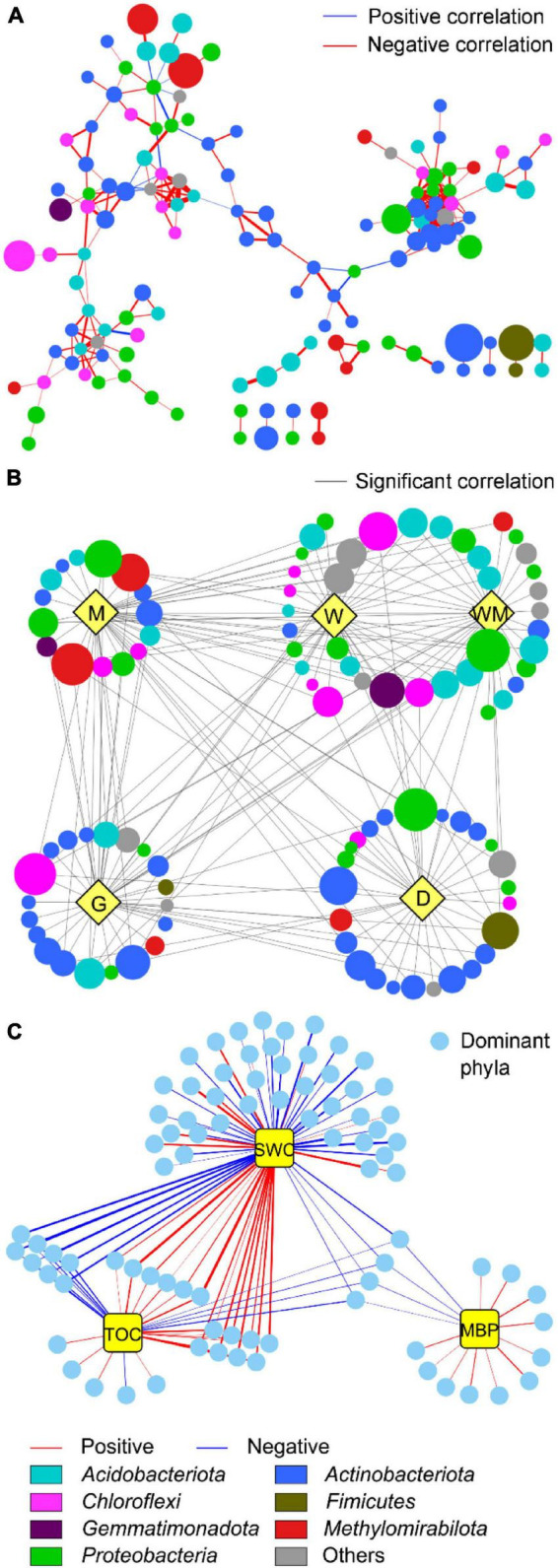
Co-occurrence networks of soil microbial communities across five wetland degradation gradients together **(A)** and individually **(B)** and their interactions with soil physicochemical properties **(C)**. The network can be organized into five groups according to the occurrence of different taxa in different gradients (indicated with yellow nodes in **(B)**: typical wetland (W), wet meadow (WM), meadow (M), grassland (G), and desert (D). Gray edges in **(B)** indicate co-occurrence of taxa in different degradation gradient groups. Nodes are colored by different dominant phyla in **(A,B)**. Yellow nodes in **(C)** indicate soil properties, including soil water content (SWC), total organic carbon (TOC), and microbial biomass P (MBP).

One-way microbial co-occurence networks across five degradation gradients (Figure 6A) had 132 nodes and 249 edges with an average number of neighbors of 3.773, an average closeness centrality of 0.52, an average degree of 3.77. A total of 38 nodes with degree higher than 3.77 and closeness centrality higher than 0.52 were obtained. The phyla *Proteobacteria*, *Acidobacteriota*, and *Actinobacteriota* were identified as keystone microbial taxa across five wetland degradation gradients. In addition, key stone taxa were transformed from *Acidobacteriota* and *Proteobacteria* in the W and WM soils into *Actinobacteriota* in the M, G, and D soils (Figure 6B). As shown in Figure 6C, SWC, TOC, and MBP had 68, 31, and 15 edges, indicating SWC possessed significant correlation with more OTUs than TOC and MBP.

## Discussion

### Microbial Community Diversity, Composition and Network Under Alpine Wetland Degradation

The lowest Shannon, Ace, and Chao indices, as well as the smallest OTU numbers, were detected in the D soils. This indicates that the degradation from typical wetland to desert reduced the microbial community richness and diversity (Table 2). Similar results were recorded in previous studies of wetland desertification (Wu et al., 2015). However, non-significant variation in the diversity indices of microbial communities was found among the W, WM, M, and G soils (Table 2). This is in contradiction with previous studies showing that wetland degradation may change microbial richness and diversity. Our findings may be attributed to the nutrient-rich environment in nearly all the soils, except for the D soils (Wu et al., 2015). Furthermore, MBP may represent microbial activity in soil (Zederer et al., 2017). Higher MBP in the WM, M, and G soils than in the W and D soils suggests that wetland degradation changed the microbial activity (Table 1).

A total of 3,163 unique OTUs in the DW soils and a range of 234–1,123 unique OTUs found in the WM, M, G, and D soils showed that wetland degradation largely shifted the microbial community compositions in the soils (Figure 2). This result was verified by the RDA diagram of this study, which showed a distinct difference in microbial community compositions along with wetland degradation and was supported by other studies focusing on wetland degradation (Figure 5; Gao et al., 2021). *Actinobacteriota*, *Acidobacteriota*, *Proteobacteria*, and *Chloroflexi* were the dominant phyla, and those taxa have also been recorded in ecosystem degradation studies conducted in coastal and swamp wetlands (Wu et al., 2021). We observed an increase in the relative abundance of *Actinobacteria* along wetland degradation gradients, which is consistent with the results conducted in the Yellow River estuary (Yu et al., 2012). The co-occurrence network (Figure 6A) shows that the main microbial phyla were interconnected for all the wetland types along the alpine wetland degradation. This result shows the high complexity of the soil community network (Layeghifard et al., 2017; Liao et al., 2020). In addition, the keystone taxa *Actinobacteriota* and *Proteobacteria* mainly contributed to the linkages within the soil microbial community networks. This is partly because of the high relative abundances of *Actinobacteriota* and *Proteobacteria* in the soil microbial community of alpine wetlands (Figure 3). As constructed by the networks for different wetland types along the alpine wetland degradation, the microbial community networks of the W soils were very similar to those of the WM soils, and *Acidobacteriota* and *Proteobacteria* were the keystone taxa in both the W and the WM soils (Figure 6B). This was verified by the non-significant separation of the W and the WM groups shown in the RDA graph (Figure 5). Most of the microbial community phyla for different wetland types were inter-connected as shown in Figure 6B (Ji et al., 2021). Moreover, *Actionbacteriota* played an important role in the microbial networks of the G and D soils, while *Acidobacteriota* were significant in the networks of the W and WM soils (Figure 6B; Ye et al., 2021; Zheng et al., 2021). The shift in keystone taxa from *Acidobacteriota* and *Proteobacteria* in the W and WM soils into *Actinobacteriota* in the M, G, and D soils may hint that these keystone taxa can be indicator for alpine wetland degradation. In conclusion, wetland degradation affected the microbial community composition as well as the microbial networks.

### Correlation Between Soil Properties and Microbial Community Compositions

SWC declined along with the wetland degradation in the study area (Table 1). This result in consistent with a previous study of a wetland degradation from peat to sand on the Zoige Plateau (Wu et al., 2015), which is mainly due to a lower water table in these ecosystems (Li Y. et al., 2021). When the typical wetland degraded to be a wet meadow, the aerobic environment increased. This enhanced the degradation of plant residues in soil and thus increased soil TOC, DOC, TP, and TN (Table 1). Similar results were previously observed (Ren et al., 2013; Li H. et al., 2021; Lin et al., 2021). The decreases in TOC, DOC, TP, and TN along with degradation from the WM and M soils to the G and D soils is mainly due to the decomposition of soil organic carbon and nutrition loss by plant uptake (Li H. et al., 2021). These results are consistent with most studies on wetland ecosystem degradation (Ren et al., 2013; Gao et al., 2021). Higher MBP in the WM, and M soils than in the W soils may result from increased soil nutrients, such as TOC, TP, and TN (Table 1; Zederer et al., 2017).

Variation in soil properties with wetland degradation may affect microbial community compositions. TOC, TP, and TN were negatively correlated with *Actinobacteria* and *Chloroflexia* and positively correlated with *Acidobacteria* (Figure 4). In addition, oligotrophic soil is not conducive to the growth of *Alphaproteobacteria* (Hu et al., 2019). The decrease in these soil properties promoted an increase in the relative abundance of *Actinobacteria* and depressed the relative abundances of *Acidobacteriota* and *Alphaproteobacteria* in the G and D soils. These soil property changes consequently affected the microbial community composition under wetland degradation (Figures 3, 5). RDA also showed that TOC, TN, and TP significantly contributed to the changes in the microbial communities along the different wetland degradation gradients (Figure 5). These observations were similar to those in a previous study showing that TOC loss induced by ecosystem development produced changes in different bacterial phyla (Li et al., 2020). Soil pH increased as the W soils degraded into the D soils (Table 1). This may explain the reduction of relative abundance of *Acidobacteriota*, which grow well in the D soils compared to the other soils (Figure 3; Hu et al., 2019). Except for *Acidobacteriota*, soil pH did not have a significant effect on the microbial communities in this study (Figure 4). SWC can have an indirect or direct influence on the abundance and diversity of soil microbial communities (Liu et al., 2018). Many studies have demonstrated that SWC may change soil microbial community composition in alpine wetlands (Wu et al., 2015; Li Y. et al., 2021). An important mechanism for the effect of SWC on soil microbial community is that SWC directly controls the oxidation-deoxidation environment, which is significant for microbial survival. The relatively anaerobic environment under high SWC conditions benefited the growth of anaerobic bacteria and suppressed the growth of aerobic bacteria (Akiyama et al., 2010). This is consistent with the positive correlations of SWC with anaerobic bacteria, such as with *Anaerolineae*, *MBNT15*, and *Methylomirabilia*, observed in this study (Figure 4). Therefore, higher relative abundances of *MBNT15* and *Methylomirabilia* were found in the W and WM soils, where SWC was higher than in the other soils (Figure 3 and Table 1). The RDA graph also revealed that SWC mostly contributed to higher relative abundances of *MBNT15* and *Anaerolineae* in the W and WM soils (Figure 5). The co-occurrence network showed that SWC possessed the most links with main microbial taxa among the soil physicochemical properties (Figure 6C; Zhan et al., 2021; Xue et al., 2022). It could be concluded that SWC appears to be a crucial factor driving the changes of microbial community compositions and networks along the gradients of alpine wetland degradation.

## Conclusion

Here we applied 16S rRNA high-throughput genome sequencing to follow the changes of microbial community compositions and networks along degradation gradients on the Tibetan Plateau. The degradation gradients started with typical alpine wetland and progressed to wet meadow, typical meadow, grassland, and desert. SWC decreased along with the process of wetland degradation. TOC, DOC, TP, TN, and MBP increased in the WM soils and then decreased along with wetland degradation from the WM to the M, G, and D soils. Wetland degradation did not affect the richness and diversity of the microbial community from the W soils to the WM, M, and G soils, but depressed richness and diversity in the D soils. Microbial community structure was strongly affected by the wetland degradation, which was driven largely by changes of soil SWC, TOC, TN, and TP. Along the wetland degradation gradients, *Actinobacteriota*, *Acidobacteriota*, *Cholorflexi*, and *Proteovacteria* closely interacted in the microbial network. Compared to the W, WM, and M soils, *Actinobacteriota* played an especially important role in the microbial co-occurrence network of the G and D soils. We conclude that degradation of alpine wetlands was strongly associated with changes in soil microbial community compositions and networks, and these were mainly influenced by SWC among those soil physicochemical properties. This research deepens our understanding of microbial community compositions and networks along the alpine wetland degradation. Based on the findings, a technique foundation for regulating specific microbial communities as affected by wetland degradation may be created. Additionally, the study may help improve alpine wetland management with the objective of maintaining the stability of alpine wetland ecosystem and preventing wetland degradation.

## Data Availability Statement

The original contributions presented in the study are included in the article/supplementary material, further inquiries can be directed to the corresponding author/s.

## Author Contributions

ML and EK investigated plant ecological diversity and collected soil samples. ML, KZ, and ZY contributed to soil characterization, statistical analysis, and data visualization. ML wrote the first draft. LL and XK improved the manuscript. All authors contributed to the article and approved the submitted version.

## Conflict of Interest

The authors declare that the research was conducted in the absence of any commercial or financial relationships that could be construed as a potential conflict of interest.

## Publisher’s Note

All claims expressed in this article are solely those of the authors and do not necessarily represent those of their affiliated organizations, or those of the publisher, the editors and the reviewers. Any product that may be evaluated in this article, or claim that may be made by its manufacturer, is not guaranteed or endorsed by the publisher.
